# Fabrication of Anisotropic Cu Ferrite-Polymer Core-Shell Nanoparticles for Photodynamic Ablation of Cervical Cancer Cells

**DOI:** 10.3390/nano10122429

**Published:** 2020-12-04

**Authors:** Shuo-Hsiu Kuo, Po-Ting Wu, Jing-Yin Huang, Chin-Pao Chiu, Jiashing Yu, Mei-Yi Liao

**Affiliations:** 1 Department of Chemical Engineering, National Taiwan University, Taipei 10617, Taiwan; b05504084@ntu.edu.tw (S.-H.K.); r05524112@ntu.edu.tw (P.-T.W.); 2 Department of Applied Chemistry, National Pingtung University, Pingtung 90003, Taiwan; mingsoo77@gmail.com (J.-Y.H.); snack95064@gmail.com (C.-P.C.)

**Keywords:** bimetallic nanoparticles, superparamagnetic nanoparticles, Fenton reaction, reactive oxygen species, cancer treatment, photodynamic therapy

## Abstract

In this work we developed methylene blue-immobilized copper-iron nanoparticles (MB-CuFe NPs) through a facile one-step hydrothermal reaction to achieve a better phototherapeutic effect. The Fe/Cu ratio of the CuFe NPs was controllable by merely changing the loading amount of iron precursor concentration. The CuFe NPs could serve as a Fenton catalyst to convert hydrogen peroxide (H_2_O_2_) into reactive oxygen species (ROS), while the superparamagnetic properties also suggest magnetic resonance imaging (MRI) potential. Furthermore, the Food and Drug Administration (FDA)-approved MB photosensitizer could strongly adsorb onto the surface of CuFe NPs to facilitate the drug delivery into cells and improve the photodynamic therapy at 660 nm via significant generation of singlet oxygen species, leading to enhanced cancer cell-damaging efficacy. An MTT (thiazolyl blue tetrazolium bromide) assay proved the low cytotoxicity of the CuFe NPs to cervical cancer cells (HeLa cells), namely above 80% at 25 ppm of the sample dose. A slight dissolution of Cu and Fe ions from the CuFe NPs in an acidic environment was obtained, providing direct evidence for CuFe NPs being degradable without the risk of long-term retention in the body. Moreover, the tremendous photo-to-thermal conversion of CuFe NPs was examined, which might be combined with photodynamic therapy (PDT) for promising development in the depletion of cancer cells after a single pulse of deep-red light irradiation at high laser power.

## 1. Introduction

Nanoparticles (NPs) have become a burgeoning alternative in biomedical use and cancer treatment because their small size allows them to exhibit an enhanced optical signal for immunosensing applications, multiple theranostics functions, and long retention time in the human body [1,2,3,4,5,6,7]. Metallic nanoparticles have drawn extensive attention and provided a new point of view on unresolved problems. The physicochemical properties of the high surface–volume ratio, localized surface plasmon resonance (LSPR) effect, and small-size-induced internalization empower the nanoparticles to approach the abnormal tissues and passively accumulate therein, improving tumor treatment efficacy [8,9,10,11,12].

Previous research has developed one-step hydrothermal synthesis to produce various metal-core–polymer-shell nanoparticles, including Au@polymer NPs, Cu@polymer NPs, and Fe_3_O_4_@polymer NPs [13]. The applications of Au@polymer NPs and Cu@Cu_2_O@polymer NPs to cancer treatment were also investigated with in vitro and in vivo experiments [14,15]. Copper nanoparticles (CuNPs) have been widely studied as a photothermal agent for near-infrared (NIR) absorption via the plasmonic effect [15,16,17,18,19,20]. When the temperature exceeds 48 °C, cells are irreversibly damaged and undergo apoptosis processes [21,22]. In addition, the better biodegradability of Cu-based nanomaterials compared to noble metals frequently used in cancer therapy makes integrating Cu element into the nanostructures less likely to accumulate in the living body [23,24,25,26,27]. On the other hand, iron-related nanoparticles are also popular candidates in biomedical applications, for example, as an effective magnetic resonance imaging (MRI) agent, bioimaging, and magnetic separation [28,29,30,31,32,33,34]. Moreover, Fe ions released from iron-related nanoparticles can enhance chemodynamic therapy by H_2_O_2_ disproportion and generate reactive oxygen species (ROS), even producing oxygen in a high H_2_O_2_ environment by Fenton reactions, a common and crucial physiological process regulating ROS levels [35,36,37,38,39]. Since cancer cells reportedly contain high levels of H_2_O_2_, an increase in ROS generation can be specific to cancer tissues [40,41]. In addition, Mazuel et al. found intracellular biodegradability of Fe-based NPs by examining the decrease in the magnetism and particle solid volume after 27 days of treatment in the tumor site [42].

Therefore, the combination of CuNPs and FeNPs with light exposure has become well-known. Liu et al. developed a CuFe_2_O_4_ nanoparticle synthesis with bovine serum albumin, proved the enhanced ROS generation ability, and examined the ability in vitro and in vivo of effective photothermal therapy (PTT) and MRI [43]. Guo et al. designed a biocompatible sub-10 nm Cu_5_FeS_4_ cube for MRI and PTT and experimented with human umbilical vein endothelial cell (HUVEC) [44]. Ding et al. verified the apoptotic cell death pathway triggered by CuFeS_2_ nanoplates with irradiation [45]. Lin et al. presented a paradigm of the facile phase transfer of hydrophobic Fe_3_O_4_@Cu_2-x_S theranostics for both MRI and PTT [46]. Comparison of the recent research studies is shown in Table 1. However, nanoparticle-mediated photodynamic therapy showed a smaller side effect to normal tissue compared with PTT treatment because the threshold to trigger photodynamic therapy (PDT) received a much lower power density threshold of laser light [3,11,12,47]. The biomedical applications of CuNP-doped copper ferrite nanoparticles to carry phototherapeutic agents and improve phototherapy are not yet well explored.

To boost the therapeutic effect, simultaneous PDT/PTT therapy has recently burgeoned [48,49,50]. Methylene blue (MB) is susceptible to light and has become one of the most prevailing photosensitizers used in conjugation with nanoparticles for cancer treatment [14,51,52]. Because of the high quantum yield of singlet oxygen species to enhance the photolysis of several cancer cell lines, MB has been loaded or encapsulated in various therapeutic nanoplatforms [53,54,55]. More importantly, MB is a drug approved by the U.S. Food and Drug Administration (FDA), supporting the feasibility of in vivo or human tests. Accordingly, it is natural to take these characteristics into account and devise an MB@NPs composite, endowing the conjoined nanoplatform with versatile advantages to attain improved performance in tumor destruction.

In this work, a copper–iron dual-metal nanocomposite (i.e., copper ferrite nanocrystal) immobilized with methylene blue for photodynamic therapy was proposed. The facile poly(styrene-alt-maleic acid) sodium salt solution (PSMA)-assisted synthesis method promised practicability in mass production of copper ferrite@PSMA nanocrystal. Due to the loaded Fe forming copper ferrite nanocrystal, the Cu-based nanoagent could trigger the ROS via Fenton reactions in the presence of an H_2_O_2_ environment. The loaded MB photosensitizer on the surface of copper ferrite nanocrystal could enhance singlet oxygen production when exposed to light at a deep red wavelength. The results substantiated this state-of-the-art copper ferrite@PSMA@MB nanocrystal design as promising for the internalization of cervical cancer cells (HeLa) and enhancing ROS generation, providing a new direction for ROS-combined phototherapy of HeLa cancer cells. Moreover, a trace of metallic Cu nanocrystals was hybridized with the copper ferrite@PSMA nanocrystal to achieve a surface plasmon resonance (SPR) band at ~600 nm. The Cu metal compartment might serve as a highly efficient light-absorbent material conducive to hyperthermia via light-to-thermal conversion in tumor sites, making it a possible candidate for photothermal therapy.

## 2. Materials and Methods

### 2.1. Materials

Copper(II) chloride dehydrate (CuCl_2_·2H_2_O), hydrochloric acid (HCl, 37%), nitric acid (HNO_3_, 65%), and Dulbecco’s modified Eagle’s medium-high glucose (DMEM/HG) were purchased from Thermo Fisher Scientific (Massachusetts, USA). Iron(II) chloride anhydrous (FeCl_2_, 99.5%), methylene blue (C_16_H_18_ClN_3_) and thiazolyl blue tetrazolium bromide (MTT) were from Alfa Aesar (Massachusetts, USA). Hydrazine hydrate (N_2_H_4_·H_2_O) and sodium pyruvate (C_3_H_3_NaO_3_S·xH_2_O) were from Acros Organics (Morris Plains, NJ, USA). poly(styrene-alt-maleic acid) sodium salt solution (PSMA), hydrogen peroxide (H_2_O_2_), 2′,7′-dichlorofluorescein diacetate (DCFH-DA), sodium bicarbonate (NaHCO_3_), trypan blue (0.4%), imidazole, N,N-dimethyl-4-nitrosoaniline (RNO), N-(3-dimethylaminopropyl)-N’-ethylcarbodiimide hydrochloride (EDC), N-hydroxysuccinimide (NHS), and folic acid (C_19_H_19_N_7_O_6_) were purchased from Sigma-Aldrich (St. Louis, MO, USA). Fetal bovine serum (FBS), trypsin-ethylenediaminetetraacetic acid(trypsin-EDTA, 0.25%) and antibiotic-antimycotic (penicillin/streptomycin/amphotericin B) were from Biological Industries (Cromwell, CT, USA). Dimethyl sulfoxide was from Scharlau (Barcelona, Spain).

### 2.2. Methods

#### 2.2.1. CuFe Nanoparticles (NPs) Synthesis

For each group, all of the reactants in Table S1 were added into a 23 mL Teflon-lined hydrothermal synthesis autoclave reactor (TM-326, TOMIN, New Taipei, Taiwan). Note that N_2_H_4_ was the last added chemical due to its high reactivity. Then the reactors were heated at 158 °C for 6 h. After heating, the reactors were placed at room temperature to cool down. The product solution was centrifuged at 11,000× *g* for 10 min, and the supernatant was then removed. The CuFe NP precipitate was resuspended in 1 mL deionized water (DI water) using an ultrasonic oscillator (DC150, Delta^®^, TM-326, TOMIN, New Taipei, Taiwan). The washing process was repeated three times. Afterward, the nanoparticle solution was centrifuged at a low speed (250× *g*) to remove large aggregations.

#### 2.2.2. Quantification of Copper and Iron Concentration

The polymer shell and the metal core had to be broken down to quantify metal concentration. 100 μL of the nanoparticle solution was first mixed with 225 μL of 12 M HCl and 225 μL of 16 M HNO_3_ to dissolve metal, followed by addition of 1800 μL of 4.5 M NaOH to break down the polymer shell. Then, 300 μL of 12 M HCl and 1350 μL of DI water were added to keep the solution acidic. Finally, the concentration of iron and copper in the nanoparticles was quantified by atomic absorption microscopy (AA; AAnalyst200, Perkin Elmer, Waltham, MA, USA).

#### 2.2.3. Metal Ratio and Optical Properties

The metal ratio was quantified by AA. The optical properties were characterized by an ultraviolet (UV)–visible spectrometer (CARY 300nc, Agilent, Santa Clara, CA, USA). The scanning region was from 800 to 200 nm, where the speed was 10 nm/s and the UV–visible slit was switched at the wavelength of 350 nm.

#### 2.2.4. Catalytic Properties of CuFe NPs

To check if CuFe NPs were able to catalyze H_2_O_2_ degradation and accelerate reactive oxygen species (ROS) generation, the DCFH-DA assay was applied. The existence of ROS could turn DCFH-DA into dichlorofluorescein (DCF), which exhibited fluorescence. 100 μL of the solution that consisted of 5 ppm of iron, 2.5 μM of DCFH-DA, and 500 μM of H_2_O_2_ was placed in a 96-well plate and reacted for 16 h. The fluorescence intensity was quantified by a multi-mode microplate reader (SpectraMax i3x, Molecular Devices, San Jose, CA, USA) at the excitation wavelength of 488 nm and the emission wavelength of 525 nm.

#### 2.2.5. Degradability Test

CuFe NPs were dispersed in DI water, phosphate-buffered saline (PBS, pH = 7.4), acidic PBS (pH = 4.5), culture medium, and 0.5% H_2_O_2_ under room temperature, respectively. At different time intervals, the CuFe NPs solutions were centrifuged at 11,000× *g* for 10 min and then the supernatant was removed. The concentration of iron and copper was quantified by AA.

#### 2.2.6. Structures, Compositions, Size Distribution and Zeta Potential

Transmission electron microscopy (TEM; JEM-2000EXII, JOEL, Tokyo, Japan) was used to determine the structure of the CuFe NPs. To characterize the composition, CuFe NPs solution was dropped on a slide glass and then vacuumed to remove water and form an opaque film. The thin film was characterized by X-ray thin-film diffractometer (XRD; X’PERT, Philips, Amsterdam, the Netherlands) at the scan speed of 0.100°/s and 0.2 s/step, where 2θ was from 20° to 80°. The size distribution and zeta potential were determined by dynamic light scattering (DLS; Zetasizer Nano, Malvern, UK).

#### 2.2.7. Magnetic Attraction

The magnetic attraction ability was simply observed by recording the solution under the magnetic field for 15 min. The observations at 0, 5, 15 min, and the side views after 15 min interaction were sorted out.

#### 2.2.8. Induced Magnetic Flux Density (B)-Magnetizing Force (H) Hysteresis Loop

The CuFe NPs solutions were frozen at −20 °C overnight, followed by lyophilization to remove water. The hysteresis loop of the CuFe NPs was measured in ±2 Tesla at 310 K with a superconducting interference magnetometer (MPMS3, Quantum Design, San Diego, CA, USA).

#### 2.2.9. Cell Culture

In this research, HeLa cells were applied in all in vitro experiments, and the cells were obtained from the Department of Plant Pathology and Microbiology, National Taiwan University. The cells were cultured in a DMEM-HG culture medium with 10% FBS and incubated in an incubator at 37 °C and 5% CO_2_. After the cells covered more than 70%, the cells were washed with PBS once. After washing, 1 mL of trypsin-EDTA was added to detach the cells. The detachment took 4 to 5 min at 37 °C and 5% CO_2_. Then, 9 mL of culture medium was added to inhibit the activity of trypsin, and the cells were transferred to a 15-mL centrifuge tube for centrifugation for 5 min at 900 rpm, 4 °C. The supernatant was removed and an appropriate amount of culture medium was added to resuspend the cells. To estimate the cell concentration, 10 μL of the cell suspension was mixed with 10 μL of trypan blue. A hemocytometer was then used to determine the concentration of viable cells.

#### 2.2.10. Cytotoxicity of CuFe NPs

HeLa cells were seeded into a 96-well plate with 100 μL of cell suspension at a concentration of 5000 cells/mL. After 24 h incubation, the cells were washed by PBS once, and 100 μL of culture medium with different metal concentrations of CuFe NPs were added. Then for another 24 h incubation, the cytotoxicity was determined by MTT assay.

The MTT colorimetric assay is based on the cleavage of MTT by viable cells. The yellow MTT molecule is reduced to purple formazan by mitochondrial enzymes. Since the formation of formazan is directly proportional to the viable cells, MTT assay can determine the relative cell number, indicating the proliferation and cytotoxicity. The relative formazan amount can be determined by reading at the absorbance of 570 nm.

After 24 h incubation, the culture medium containing NPs was removed, and the cells were washed by PBS once. The MTT working stock was diluted by 10 folds with culture medium as 0.5 mg/mL. 100 μL of culture medium containing MTT was added, and the cells then reacted with MTT for 3.5 h at 37 °C and 5% CO_2_. Afterward, the medium was removed and 100 μL of DMSO was added to dissolve formazan produced by viable cells. After shaking for 30 min avoiding light, the absorbance at 570 nm was measured by a microplate reader. The blank absorbance was defined as the absorbance of the group with no cells but treated with MTT culture medium for 3.5 h and then replaced by 100 μL DMSO. The activity of cells was defined as the ratio between the NP-treated groups and the non-NP-treated group.

#### 2.2.11. Temperature Elevation of CuFe NPs

CuFe NPs were first diluted with DI water to 100 ppm metal. 100 μL of CuFe NPs solution was added into a 96-well plate and then exposed to red laser at the wavelength of 660 nm. The temperature of the solution was detected by a digital thermometer with a thermocouple probe every 30 s and the temperature elevation was observed.

#### 2.2.12. Methylene Blue Loading and Purification

We added 3 mM methylene blue (MB) solution dropwise into 1 mL CuFe NPs solution and the mixture was rotated avoiding light for 18 h for MB absorption. To remove excess MB, the mixture was centrifuged at 11,000× *g* for 10 min and the supernatant was collected for quantification. Using UV–visible spectroscopy (CARY 300nc, Agilent, Santa Clara, CA, USA), the MB calibration curve was first established, and the corresponding amount of MB absorbed could be estimated. Then, the precipitation was redispersed with 1 mL of DI water under ultrasonic oscillation. The purification step was repeated three times, and the NP solution was finally redispersed and stored in 1 mL DI water for further use.

#### 2.2.13. Detection of Reactive Oxygen Species from Methylene Blue (MB)-CuFe NPs after Irradiation

We transferred 1 mL of the as-synthesized methylene blue-loaded CuFe NPs (abbreviated as MB-CuFe NPs) solution into a 1.5 mL Eppendorf tube. 2 μL of 12.5 mM N,N-dimethyl-4-nitrosoaniline (RNO) and 10 μL of 20 mM imidazole were added, and the mixture was vortexed for homogeneous mixing. Then the mixture was irradiated with 660 nm laser at 75 mW/cm^2^ for 10 min, where the distance between the liquid surface and the light source was about 1 cm. Afterward, the Eppendorf tube was covered with aluminum foil to avoid light for further UV–visible examination.

#### 2.2.14. Dark Toxicity of the MB-CuFe NPs

To examine dark toxicity, HeLa cells were seeded into a 96-well plate at a concentration of 5000 cells/mL. The MB-CuFe NPs solution was first centrifuged to remove DI water and resuspended in culture medium under ultrasonic oscillation. By serial dilution, different concentrations were obtained. After 24 h incubation, the cells were washed by PBS once, and 100 μL of culture medium containing different metal concentrations of CuFe NPs were added. Then, for another 24 h incubation, the cytotoxicity was determined by MTT assay. The control group was the group that was not co-incubated with nanoparticles.

#### 2.2.15. Detection of In Vitro Reactive Oxygen Species Generation

HeLa cells were seeded in a 24-well plate (12,000 cells/well) and incubated for 24 h. After removing the medium, the cells were washed with PBS once. 1 mL of culture medium containing different metal concentrations of MB-CuFe NPs was added and was co-incubated with the cells for 24 h at 37 °C and 5% CO_2_. After removing the medium, the cells were washed with PBS once. DCFH-DA, a fluorogenic reagent, was used to measure the generation of reactive oxygen species (ROS). The DCFH-DA stock was diluted in culture medium to 20 μM. Each well was treated with 1 mL DCFH-DA-containing medium. After incubating for 30 min, the wells were irradiated with a 660 nm laser at 75 mW/cm^2^ for 10 min. Followed by another 60 min incubation, the reagent was removed and the cells were washed with PBS once. The fluorescence exhibited was observed by fluorescence microscopy, and the fluorescence intensity was quantified by the software Image J (U. S. National Institutes of Health, Bethesda, MD, USA).

#### 2.2.16. Cell Activity before and after Nanoparticle Treatment under Light Irradiation

After the synthesis of MB-CuFe NPs, folic acid was conjugated to improve accumulation at the targeted cells. 4 mg EDC and 4 mg NHS were separately dissolved in 400 μL of DI water and then mixed. 200 μL of this EDC/NHS solution was afterward mixed with 600 μL of DI water and 50 μL of 0.05 M folic acid, followed by sonication for 5 min avoiding light. Then, 200 μL of the NP sample solution was added to the previously prepared mixture. It was sonicated for 60 min under an ice bath, centrifuged for 10 min to remove supernatant, and resuspended with culture medium for use. Different metal concentrations were prepared by serial dilution. HeLa cells, which had been seeded and incubated for 24 h at the density of 12,000 cells/well in a 24-well plate, were treated with the medium of different metal concentrations for 4 h. After 4 h, the medium was removed, and the cells were rinsed with PBS once and refreshed with non-NP-containing medium. Then, each well was irradiated with 660 nm laser at 75 mW/cm^2^ for 10 min. After irradiation, the cells were incubated for another 24 h. Lastly, the cell activity was determined by MTT assay to compare between the results before and after light treatment.

#### 2.2.17. Statistical Analysis

All data were expressed as means ± standard deviation. A comparison of different groups was determined using one-way analysis of variance (ANOVA) and a significant difference was assumed at *p* value ≤ 0.05.

## 3. Results

### 3.1. Research Outcomes

#### 3.1.1. Characterization of CuFe NPs

**Figure 1:**
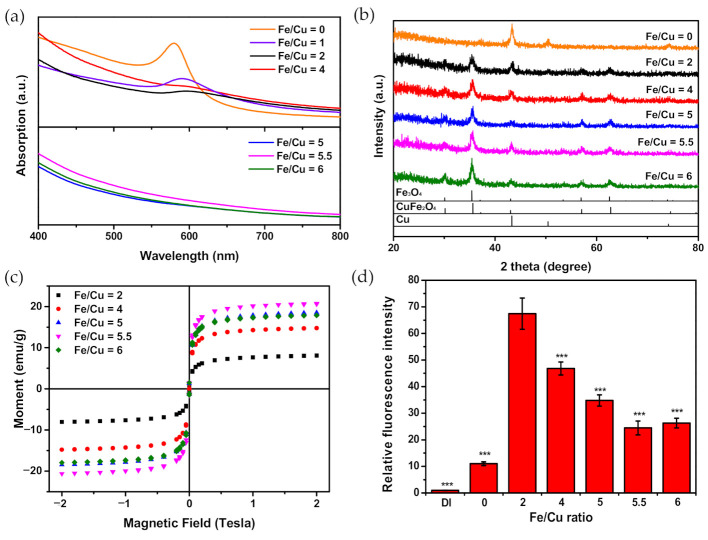
(**a**) UV–visible spectrum and (**b**) X-ray diffraction (XRD) pattern of CuFe nanoparticles (NPs). (**c**) Hysteresis loops of CuFe NPs with different Fe/Cu ratios. (**d**) Quantification of H_2_O_2_ catalytic ability by 2′,7′-dichlorofluorescein diacetate (DCFH-DA, *n* = 4. *** *p* < 0.001, compared to Fe/Cu ratio of 2).

Figure 1a shows the UV–visible absorption spectrum of CuFe NPs with different Fe/Cu ratios. For groups with lower Fe/Cu ratios (namely, 0–4), absorption peaks at the wavelength between 580 nm and 600 nm were obtained, meaning the LSPR behavior of nano-sized copper appeared [56,57,58]. As the Fe/Cu ratio went higher than 5, these peaks were no longer observable in groups. An absorbance peak with an Fe/Cu ratio equal to 2 slightly shifted to 660 nm, while the Fe/Cu = 0 group had an absorbance peak at 580 nm. The redshift wavelength is close to the NIR-I wavelength window. Although the Fe/Cu of CuFe NPs at 2 provided significant benefit for NIR light absorption, the extinction at 580–600 nm remarkably decreased. Thus, the subsequent PDT treatment with the lower power threshold did not show a photothermal injury without side effects by heat during a long PDT reaction period.

The composition of metallic Cu and ferrite structure in the CuFe NPs was determined by taking XRD measurements (Figure 1b). In the Cu only group (Fe/Cu ratio of 0), the pure Cu nanoparticle was produced without the ferrite impurity’s reflection peaks. When including 6.21 mM Fe (Fe/Cu ratio of 2), the XRD spectrum revealed the new reflection pattern related to the cubic spinel structures of Fe_3_O_4_ and/or CuFe_2_O_4_ [59,60]. Note that the reflection peak intensity of 2θ at 50.43° decreased with the increased iron reagent concentration from Fe/Cu at 0 to Fe/Cu at 4. Therefore, the absorbance decline at 580–660 nm was attributed to the decreased Cu population in CuFe NPs.

After the synthesis and purification processes were complete, the CuFe NPs were well dispersed in DI water. The copper and iron concentrations of each group were quantified by atomic absorption. As shown in Figure S1a, the Fe concentration of NPs increased as the Fe/Cu ratio of the reactant increased. In the groups with a higher Fe/Cu reactant ratio, the product’s Fe concentration slightly decreased. In contrast, the copper concentration declined as the Fe/Cu ratio in the reactant increased (Figure S1b). Since Fe’s initial concentration manipulated the Fe/Cu ratio of nanoproducts, the decreased Cu concentration could be attributed to the increase in Fe ions competing for reduction. Figure S1c shows the Fe/Cu molar ratio. Although the Fe concentration of the nanoproducts decreased when the reactant ratio was over 5, the Fe/Cu molar ratio still increased. The decrease of the metallic Cu crystal in the XRD measurement (Figure 1b) appeared to be due to the dissolution and release in the solution, which could allow the Cu ion source into the copper ferrite lattice. However, we observed that the decrease in Cu concentration in the product resulted in inconsistent Fe/Cu ratios in the batch-prepared products.

In addition, TEM images were used to analyze these CuFe NPs, as shown in Figure S2, where it can be seen that a dark contrast appeared in the center, showing the inorganic composites embedded in the light-contrasting PSMA polymer. Such a composition was similar to the previous core-shell structure. As the amount of FeCl_2_ added increased in the same reaction, the single inorganic core in the particles was converted into a multi-core nanostructure with a spindle-like shape. Moreover, the polymer encapsulating inorganic nanocores had a higher yield with the increment of the loading iron concentration. These CuFe NPs were oval, as shown in the TEM images. However, the shape of the CuFe NPs became irregular and turned to aggregates when the Fe/Cu ratio increased to 6. According to the statistics, the average diameter was 66.04 ± 9.65, 70.99 ± 11.75, 115.89 ± 34.37, 97.87 ± 30.67, 118.37 ± 34.90, and 90.57 ± 27.04 nm for Fe/Cu ratios of 0, 2, 4, 5, 5.5, and 6 respectively (Figure S3). Moreover, the average core diameter was 21.33 ± 9.24 nm for the single-core structure, indicating the average thickness of the PSMA shell was 22.35 ± 13.36 nm.

To characterize the colloidal dispersivity of CuFe NPs in aqueous phase, DLS was applied to determine the hydrodynamic diameter and zeta potential (Table S2). The polydispersity index (PdI) of each group was lower than 0.3, representing the uniformity of the CuFe NPs. The zeta potential determined the negatively charged surface property of the CuFe NPs to be around −30 mV, indicating the exposure of carboxylate groups at PSMA polymer in the inorganic core-polymer shell structure. A considerable charge value could provide enough electrostatic repulsion to aid the colloid dispersion in the aqueous solution.

#### 3.1.2. Magnetic Attraction

**Figure 2:**
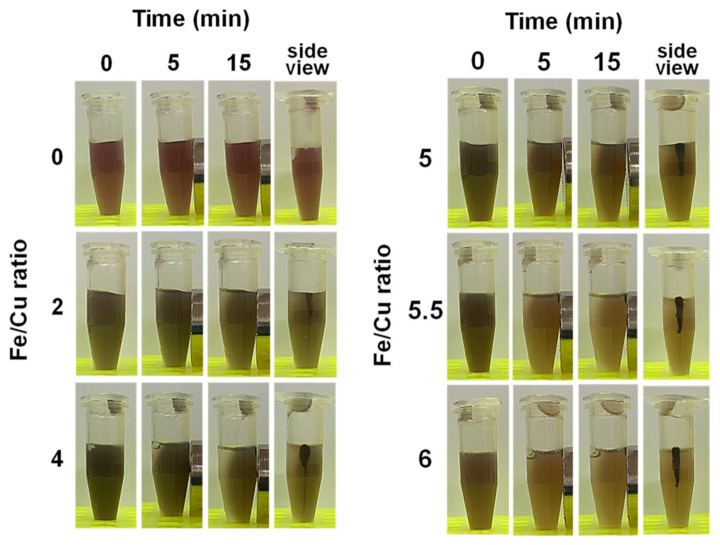
Photos of CuFe NPs under magnetic field after 0, 5, 15 min and the side view of CuFe NPs under magnetic field after 15 min.

To examine whether CuFe NPs are able to respond to a magnetic field, a cylinder-shaped neodymium magnet was used to attract the as-prepared CuFe NPs (Figure 2). For the groups with Fe, CuFe NPs gathered at the magnet within 15 min. The aggregation could also be resuspended in DI water by 3-s vortexing. The group with an Fe/Cu ratio of 0 showed no response to the magnetic field because there was weak magnetization of Cu in the crystal. However, the appearances of different groups showed no clear difference. Further magnetic properties were characterized by a B-H Curvehysteresis loop. The hysteresis loops of CuFe NPs with different Fe/Cu ratios were measured by a superconducting interference magnetometer (Figure 1c). The saturation magnetization value increased with the Fe/Cu ratio, which was consistent with the high magnetization of Fe combined in the ferrite crystals. The saturation magnetization values of groups of the Fe/Cu ratio were 8.14 emu/g at 2, 14.77 emu/g at 4, 18.53 emu/g at 5, 20.75 emu/g at 5.5, and 17.88 emu/g at 6. Note that the decreased magnetization occurred at an Fe/Cu ratio equal to 6 because of the possible increase in the antiferromagnetic property in the ferrite crystal host. In addition, the magnetic loops lacked remanent magnetization for all the CuFe NP samples. The intersection of the x-axis, representing the coercivity, was also smaller than 10 Oe, exhibiting a typical superparamagnetic behavior. It has been reported that superparamagnetic nanomaterials can align the magnetic moment from the dispersion phase to the targeted direction in the lesion area when the magnetic field-guiled operation is performed. Furthermore, the feasibility of superparamagnetic nanoparticles with strong magnetization serving as an MRI agent was already proven in several studies in vitro or in vivo [37,61,62].

#### 3.1.3. Enhanced Conversion of H_2_O_2_ to ROS with CuFe NPs

H_2_O_2_ is an indispensable intermediate in cell death, generating reactive oxygen species (ROS) to induce the apoptosis process. The DCFH-DA molecule was utilized to quantify the conversion of H_2_O_2_ to generate ROS with CuFe NPs. CuFe NPs, acted as catalyst, could perform a Fenton-like catalysis reaction. Figure 1d shows the relative intensity of the DCF fluorescence. As the Fe/Cu ratio decreased from 6 to 2, increasing Cu concentration, the fluorescence intensity increased. However, the group with Fe/Cu ratio of 0, consisting of the only Cu, produced weak fluorescence, indicating low conversion efficiency from H_2_O_2_ to ROS. The result indicated that the combination of Fe with Cu in the oxide form could enhance the catalytic ability to generate ROS [63,64,65]. The optimal Fe/Cu ratio of 2 was shown to have reached the highest conversion rate.

#### 3.1.4. Degradability Test

To quantify the degradability of CuFe NPs, the nanoparticles were dispersed in five different kinds of solvent, including PBS (pH = 7.4), acidic PBS (pH = 4.5), DI water, culture medium, and 0.5% H_2_O_2_. The nano-precipitates were centrifuged and collected at different time intervals, and the metal concentrations from the separated supernatant were quantified. As shown in Figure S4, it was found that the Fe ions of the samples did not degrade in any solvent, where the remaining Fe concentration was around 100%. The dissolution of Cu ions from the CuFe NPs was not determined in DI water and neutral PBS. However, we found that the Cu ions were dissolved in acidic PBS within 8 h (Figure S5). It was proposed that the nano-sized Cu species could react with H+. The dissolution proportion varied with the Fe/Cu ratio; it was 23.29% at 0, 46.34% at 2, 64.52% at 4, 85.00% at 5, 87.50% at 5.5, and 86.67% at 6. In addition, most groups reach the maximum amount of dissolution within 8 h, whereas the group with an Fe/Cu ratio of 4 displayed slightly different behavior, where the remaining copper percentage continued to drop after 8 h and the dissolution proportion reached 77.42% after 16 h. Similarly, Cu was dissolved in the culture medium. This result could be attributed to the salt, proteins, antibiotics, and other substances in the culture medium, which might react with Cu and lead to ionization.

#### 3.1.5. Cytotoxicity

The cytotoxicity of CuFe NPs was quantified by MTT assay after 24 h co-incubation with HeLa cells (Figure S6). Compared to the group with a Fe/Cu ratio of 0, all the other groups exhibited significantly lower cytotoxicity from 2 ppm to 100 ppm (all *p*-values < 0.001). The cell viability rose as the Fe/Cu ratio increased, corresponding to a decreased Cu proportion in CuFe NPs. For CuFe NPs containing Fe, the HeLa cells’ cell viability was not influenced when the metal concentration was under 5 ppm. The cell activity of all groups with Fe was over 60% at a concentration of 50 ppm.

Although the group with an Fe/Cu ratio of 2 had the most significant H_2_O_2_ catalytic ability (Figure 1d), the CuFe NPs showed much-improved cell viability when compared with the group with an Fe/Cu ratio of 0 (Figure S6). When the concentration exceeded 5 ppm, toxicity was observed and increased with concentration. The cell activity of all groups with Fe was over 75% at a concentration of 0–25 ppm. Based on the dose-dependent results, the low dose of this CuFe NP was selected for further experiments.

#### 3.1.6. Temperature Elevation

**Figure 3:**
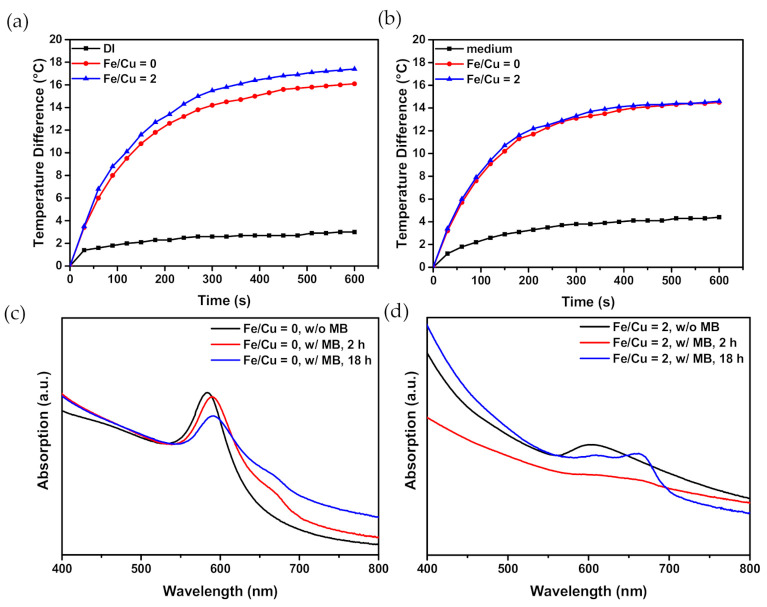
(**a**) The photothermal effect of CuFe NPs at 100 ppm metal with Fe/Cu ratio of 0 and 2 in DI water or in (**b**) culture medium. (**c**) The UV–visible spectrum of methylene blue (MB)-CuFe NPs of Fe/Cu ratio of 0 and (**d**) 2 at different reaction times. Note that MB has an absorbance peak at a wavelength of 660 nm.

Photothermal therapy is based on the characteristic that cells undergo irreversible death processes when the temperature exceeds 48 h. The temperature elevation of groups with Fe/Cu ratios at 0 and 2, at a metal concentration of 100 ppm, were measured and are shown in Figure 3a,b. Both groups in DI water elevated the temperature by at least 10 °C higher than DI water only, and the group with an Fe/Cu ratio of 2 showed higher temperature elevation efficiency. The result that the group with an Fe/Cu ratio of 2 could contribute to a larger temperature difference than that at 0 is worth noting. The measurement of temperature elevation was based on the same metal concentration, meaning the Cu concentration of the group with an Fe/Cu ratio of 2 was only 20% of that of the group with Cu only. Since the extinction coefficient of Cu is greater than that of Fe, the temperature elevation of the group with Cu only was expected to be higher [15,29,66]. However, the result was the opposite, meaning that Fe could assist with Cu’s temperature elevation. The temperature elevation in the culture medium was also measured. The behaviors of the three tested groups were similar but showed slightly lower temperature differences than those in DI water because the substances in the culture medium might absorb light and poorly transfer the energy into heat. The temperature elevation of the culture medium only being higher than that of DI water also confirmed the assumption.

#### 3.1.7. Structures and Optical Properties of MB-Immobilized CuFe NPs

**Figure 4:**
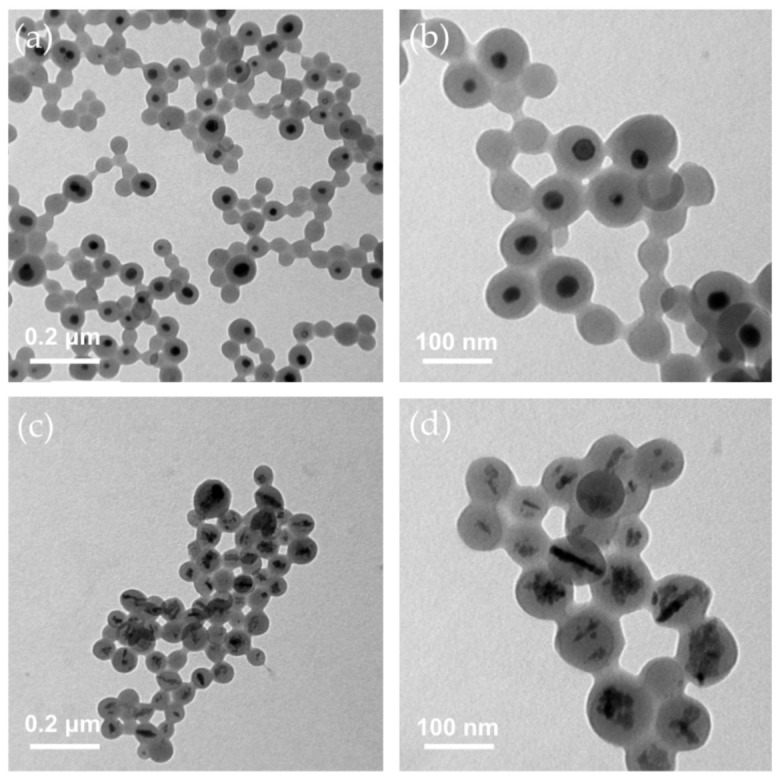
Transmission electron microscopy (TEM) images of CuFe NPs. (**a**,**b**) Fe/Cu ratio of 0 shows a single-core structure. (**c**,**d**) Fe/Cu ratio of 2 shows a multi-core structure.

According to previous examinations, an Fe/Cu ratio of 2 was chosen as the experimental group on MB immobilization and its extended properties, whereas Fe/Cu ratio of 0 was taken as the control group. After immobilizing methylene blue (MB), the TEM images showed no obvious change in morphology compared to the CuFe NPs (Figure 4). This result provided direct evidence that MB immobilization did not destroy the NP structure, as demonstrated by the lack of destruction at the surface and the core area of CuFe NPs.

As shown in Figure 3c,d, the UV–visible spectrum of the MB-immobilized CuFe NPs exhibited an obvious peak at 660 nm, while CuFe NPs did not possess specific peaks. MB has the strongest characteristic absorption peak at 660 nm. Both results demonstrated the successful immobilization of MB onto the surface of CuFe NPs. Moreover, our tests showed that MB loading efficacy was higher after an 18-h incubation time than that under the same reaction with a 2-h incubation.

#### 3.1.8. Size Distribution, Zeta Potential, and Drug Loading Content of MB-Immobilized CuFe NPs

**Figure 5:**
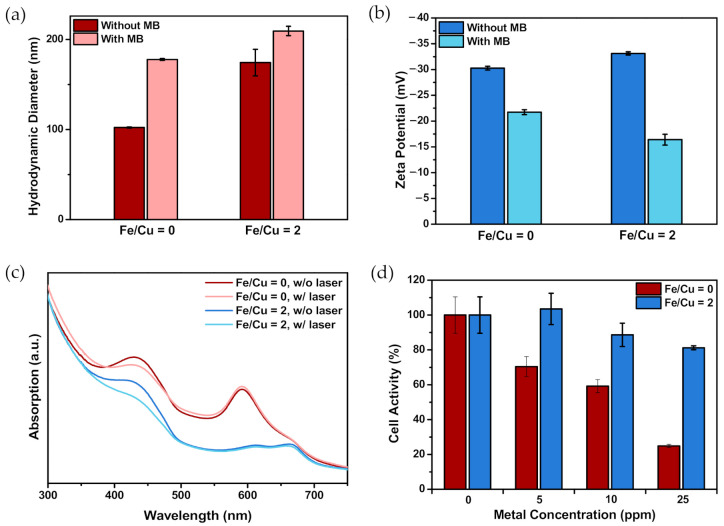
(**a**) Hydrodynamic diameter and (**b**) zeta potential of MB-CuFe NPs before and after MB conjugation. (**c**) The UV–visible spectrum of the N,N-dimethyl-4-nitrosoaniline (RNO)/imidazole-treated MB-CuFe NPs solution before and after laser irradiation for 10 min. The peak at 440 nm indicates the existence of RNO reagent, which can be degraded by reactive oxygen species (ROS). (**d**) Cell activity of cervical cancer HeLa cells after 24 h co-incubation with MB-CuFe NPs. Over 80% of the cells are viable at a metal concentration of 25 ppm in the group with an Fe/Cu ratio of 2.

Following MB conjugation, the average hydrodynamic diameter slightly increased in both groups, suggesting MB’s existence on the surface of the NPs (Figure 5a). To further examine this assumption, the zeta potential was measured. After the reaction, the zeta potential for the groups with Fe/Cu ratios of 0 and 2 increased from −30.27 and −33.13 mV to −21.73 and −16.40 mV, respectively (Figure 5b). Since MB is positively charged, the results confirmed the successful conjugation of MB. As for the drug loading content, the amount of MB loaded was also assessed. The MB concentration in the final product was calculated to be 30–50 μM at a metal concentration of 25 ppm, which was high enough for photodynamic therapy.

#### 3.1.9. Detection of Reactive Oxygen Species after Irradiation

RNO shows a pronounced absorption peak at 440 nm, but the peak declines if reactive oxygen species are generated [53,67]. Figure 5c clearly showed a significant decrease in absorbance at 440 nm as a function of irradiation time after 660 nm laser irradiation. This result suggests that MB-CuFe NPs could induce ROS generation, thereby being a potential photodynamic therapy agent.

#### 3.1.10. Dark Toxicity of CuFe NPs

The MTT assay was applied to evaluate the cell activity of HeLa cells after being co-incubated with MB-CuFe NPs for 24 h (Figure 5d). Compared to the group with an Fe/Cu ratio of 0, the group with an Fe/Cu ratio of 2 showed a much higher metabolic activity, and the cell activity remained above 80% at a metal concentration of 25 ppm. Since Cu is less biocompatible, under the same metal concentration, the group with an Fe/Cu ratio of 0 had a higher proportion of Cu and would induce higher toxicity.

#### 3.1.11. In Vitro Reactive Oxygen Species Generation

**Figure 6:**
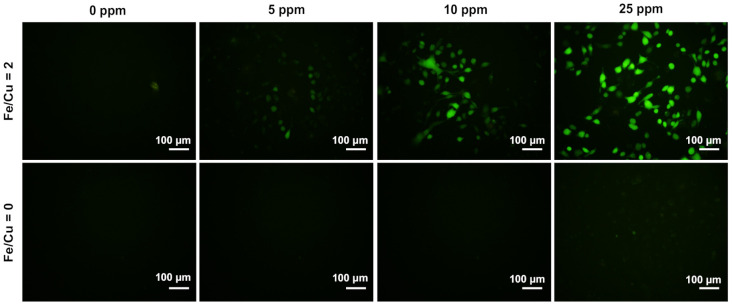
DCFH-DA fluorescence performance at different metal ratios and different metal concentrations. The stronger fluorescence signal indicates more ROS are generated. (Scale bar: 100 μm).

**Figure 7:**
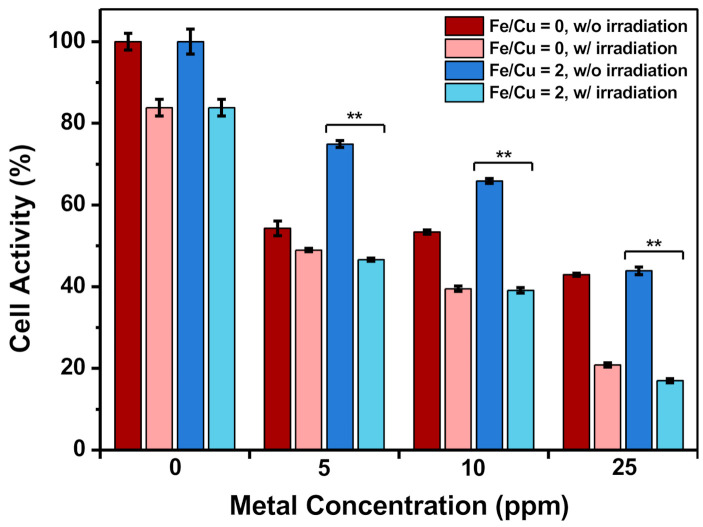
Cell activity of HeLa cells with and without laser irradiation. (** *p* < 0.01; MB-CuFe NPs co-incubation time: 4 h).

Different metal concentrations under 25 ppm in both groups were applied in the in vitro experiments. HeLa cells were first co-incubated with the MB-immobilized CuFe NPs for 24 h, washed with PBS, and then refreshed with DCFH-DA-containing medium for another 30 min. Afterward, the cells were irradiated with laser for 10 min, further incubated for 60 min, and then observed with fluorescence microscopy to determine the ROS generated in cells. Figure 6 shows that the fluorescence was barely detected in the group with an Fe/Cu ratio of 0. In contrast, the fluorescence signal was more significant in the group with an Fe/Cu ratio of 2, even at a low metal concentration of 5 ppm. As the metal concentration increased, the DCF performance also improved. This may be attributed to the higher MB concentration, which would induce more ROS generation after irradiation. Furthermore, the MTT results showed significant differences before and after laser irradiation (Figure 7). Since the cells were co-incubated with the nanoparticles for 4 h only and showed lower activity after irradiation, it was inferred that the cells might take in the nanoparticles through endocytosis. With the help of laser irradiation, the endocytosed nanoparticles could spark the generation of more ROS within the cells, therefore accelerating the progress of reaching the apoptosis phase and finally leading to cell death.

## 4. Discussion

Dual-metal nanoparticles encapsulated into PSMA polymer layer was developed to perform a combined optical and magnetic function in this work. The hydrothermal reaction of FeCl_2_, N_2_H_4_, and PSMA has been demonstrated to fabricate Fe_3_O_4_@PSMA nanoparticles by Huang and co-workers [13]. Because of the similar lattice constant between Fe_3_O_4_ and CuFe_2_O_4_ crystal [58,59], the incorporation of Cu ions into the spinel structure was allowed to generate high crystalline nanoparticles. The formation of the anisotropic nanostructure of CuFe NPs can be attributed to the kinetic control crystallization process [68] relying on the mixture of Cu ions into the iron oxide crystal by dissolution of Cu NPs (Figure 4a,b) rather than direct co-precipitation reaction upon the simple mixture of Fe and Cu ions in a base condition [43,69].

Although the group with an Fe/Cu ratio of 2 had the most significant H_2_O_2_ catalytic ability (Figure 1d), the CuFe NPs showed much-improved cell viability when compared with the group with an Fe/Cu ratio of 0 (Figure S6). When the concentration exceeded 5 ppm, toxicity was observed and increased with concentration. The cell activity of all groups with Fe was over 75% at a concentration of 0–25 ppm. Based on the dose-dependent results, the low dose of this CuFe NP was selected for further experiments.

It has been reported that hydrogen peroxide plays an important role in cancer development, where H_2_O_2_ was found to be highly produced by cancer cells [40]. Several reports presented ROS-induced chemodynamic therapy as being strongly evolved from the cancer cells in the presence of the iron-based nanoparticles [43,70,71,72]. The chemodynamic effect is commonly inferior to normal cells because the malignant cells possessed high intracellular H_2_O_2_ levels compared with normal cells. As a proof-of-concept, in our work, the DCF was utilized to evaluate the potential application of the Fenton-like catalysis reaction with CuFe NP catalyst to generate toxic ROS by converting the intracellular H_2_O_2_ (Figure 1d and Figure 6). Following optimization of the Fe/Cu ratio, an FDA-approved photosensitizer, methylene blue (MB), was conjugated with CuFe NPs to improve PDT efficacy. The surface charge discrepancy confirms the adsorption of MB onto the surface of CuFe NPs (Figure 5b). Compared to previous reports, the optimal MB concentration in our work is considered high enough to improve PDT efficacy [73,74]. After 10 min of 660 nm irradiation, the in vitro results showed that the efficient PDT killed over 40% more HeLa cells at the sample dose of 5 ppm, compared to the non-irradiated group (Figure 6 and Figure 7). Although several works have focused on the iron–copper composite Fenton catalysts [75,76], the use of highly dispersed anisotropic CuFe NPs for enhancing the conversion of H_2_O_2_ to bio-stimulate cancer cells and combined with PDT has not been reported.

Noble metal nanocomposites, such as Ag/Au nanocage, spiky star-shaped Au/Ag NPs and Ag/Nd NPs, were proposed as potential materials for photothermal therapy [77,78,79]. However, the Au alone and Ag-based NPs lacked the magnetic property for an imaging application of MRI [80] and could not efficiently generate ROS via the conversion of H_2_O_2_ molecules. Because the photothermal effect may contribute an additional heat transport process to harm the cells and induce endogenous H_2_O_2_, CuFe NP was promising in further PTT/PDT therapeutic strategies. It is noteworthy that the anisotropic CuFe NPs increased their particle size in length (Figure 4c,d and Figure S2) and thus were suitable and conducive to the enhanced permeability and resonance (EPR) effect by passively targeting solid tumor tissues [81,82,83,84]. In addition, the Cu/Fe composite in the CuFe NPs was degradable (Figure S5) to possibly prevent long-term retention and improve the biocompatibility for the potential photomedicine development.

## 5. Conclusions

In summary, we devised copper–iron bimetallic nanoparticles loaded with methylene blue (MB-CuFe NPs) for photodynamic/photothermal combined therapy. Through the one-step hydrothermal reaction, the facile synthesis process allows the mass production of the CuFe NPs. The incorporation of Fe not only causes a redshift in the UV–visible spectrum but also renders the nanoparticle superparamagnetic. Besides, the outstanding H_2_O_2_ catalytic ability further improves ROS generation. Therefore, CuFe NPs serve not only as a photocatalyst but also as a possibly effective photothermal agent and even a potential bioimaging resource, while MB enhances ROS generation and facilitates in vitro PDT therapeutic effects. To our knowledge, this study on CuFe NPs hybridized with MB could be a further development of MRI-guided combined phototherapy, which may shed light on a new concept of cancer treatment.

## Figures and Tables

**Table 1:** Comparison of relevant works on copper and iron nanocomposites.

Author	Material	Application	PTT	MRI	Degradability	ROS Enhancement
Liu et al.	CuFe_2_O_4_	Cancer therapy	**✓**	**✓**		**✓**
Wang et al.	Cu_5_FeS_4_	Cancer therapy	**✓**	**✓**		
Tai et al.	Cu@Cu_2_O	Cancer therapy	**✓**		**✓**	**✓**
Kao et al.	Fe_3_O_4_	Atherosclerosis diagnosis		**✓**		
Mazuel et al.	γ-Fe_2_O_3_	Intracellular biodegradation		**✓**	**✓**	
Lin et al.	Fe_3_O_4_@Cu_2−x_S	MRI, cancer therapy	**✓**	**✓**		

## Supplementary Materials

The following are available online at https://www.mdpi.com/2079-4991/10/12/2429/s1, Figure S1: Metal ratio and UV-Vis spectrum of CuFe NPs, Figure S2: TEM images of CuFe NPs with different metal ratios, Figure S3: Statistics of the average diameter of CuFe NPs with different metal ratios, Figure S4: Relative remaining Fe of CuFe NPs after 16 h dispersion in different solvents at 25 °C, Figure S5: Relative remaining Cu of CuFe NPs after 16 h dispersion in different solvents at 25 °C, Figure S6: Cell activity of HeLa cells after 24 h co-incubation with CuFe NPs, Table S1: Formula of synthesis of CuFe NPs, Table S2: Hydrodynamic diameter and zeta potential of CuFe NPs.
