# Supplementary material for: Fabrication of Anisotropic Cu Ferrite-Polymer Core-Shell Nanoparticles for Photodynamic Ablation of Cervical Cancer Cells
Source: Nanomaterials (Basel). 2020 Dec 4;10(12):2429. doi: 10.3390/nano10122429 (PMC7761902; doi:10.3390/nano10122429)
Supplement: Supplementary file 1 [file nanomaterials-10-02429-s001.pdf]

nanomaterials

MDPI

Supplementary Information

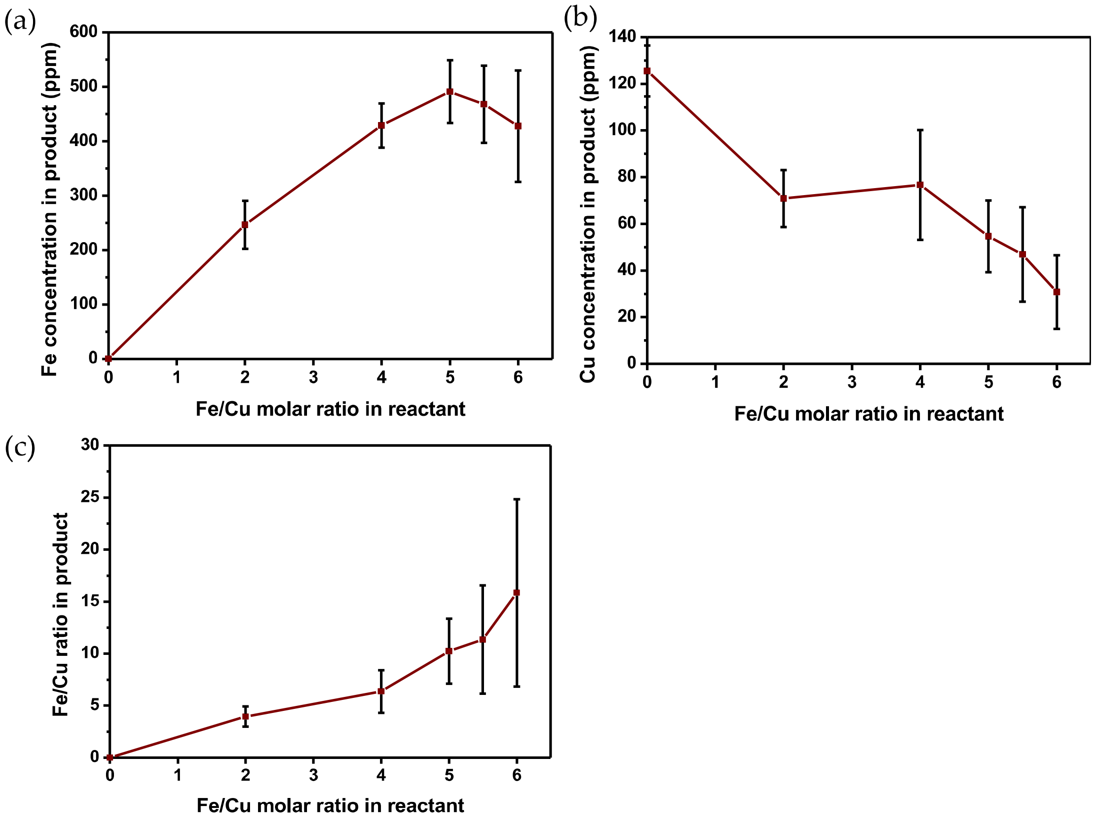

Figure S1. Metal ratio and UV-Vis spectrum of CuFeNPs. (a) Fe concentration of CuFeNPs. (n = 8) (b) Cu concentration of CuFeNPs. (n = 8) (c) Fe/Cu ratio of CuFeNPs. (n = 8)

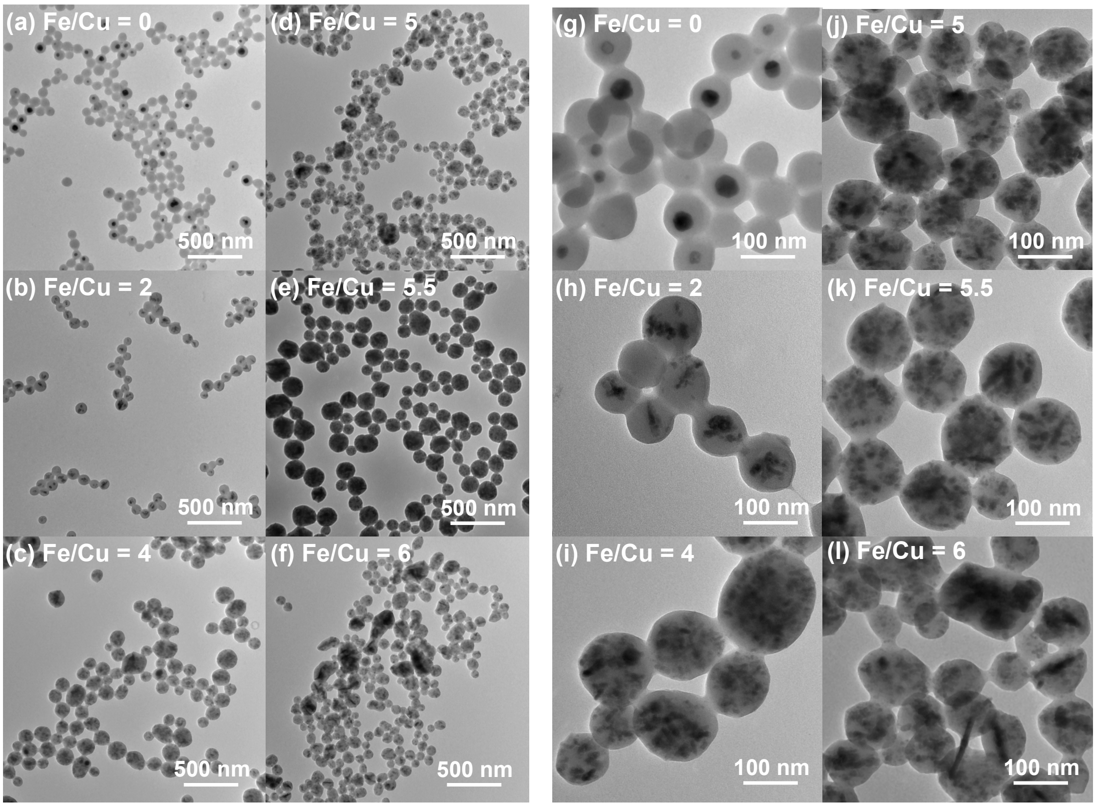

Figure S2. TEM images of CuFeNPs with different metal ratios. Scale bar: (a–f) 500 nm, (g–l) 100 nm.

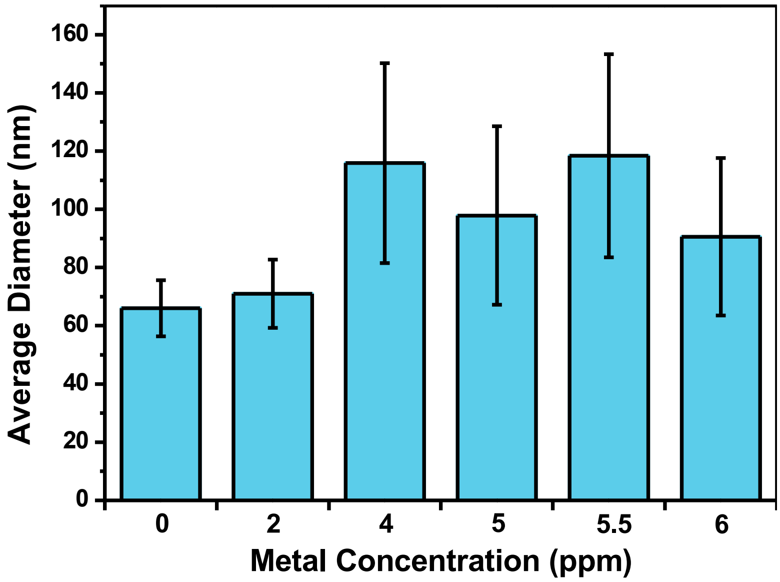

Figure S3. Statistics of the average diameter of CuFeNPs with different metal ratios.

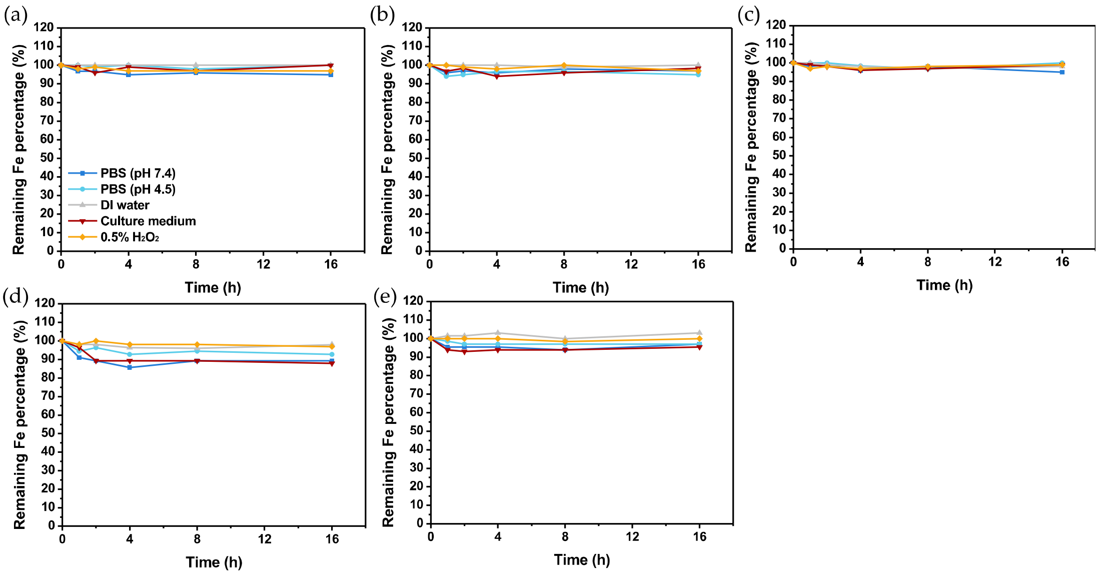

Figure S4. Relative remaining Fe of CuFeNPs after 16 h dispersion in different solvents at 25°C. (ae) Fe/Cu ratio at 2, 4, 5, 5.5 and 6, respectively.

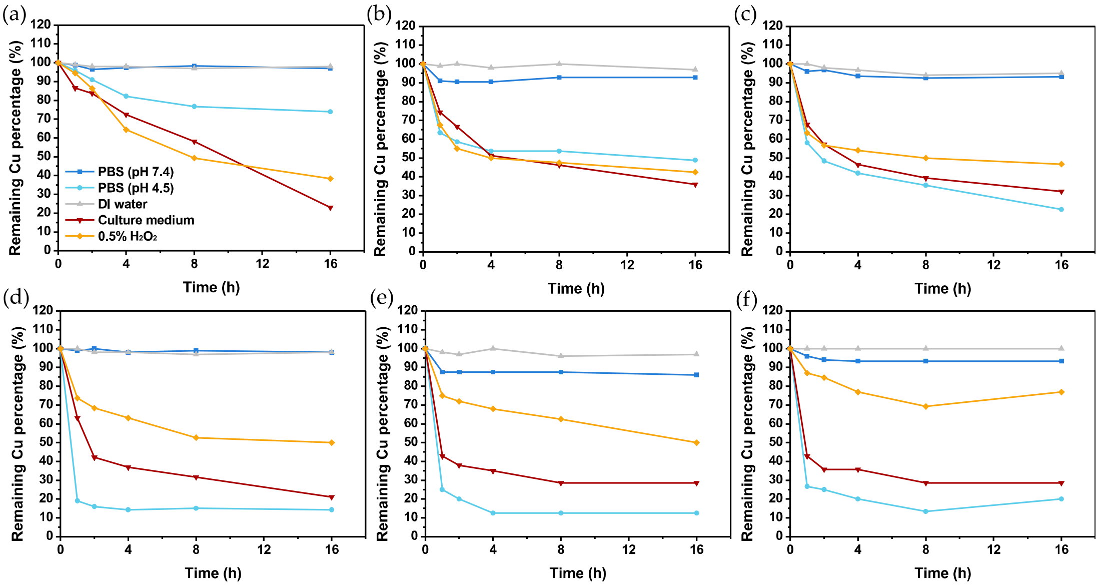

Figure S5. Relative remaining Cu of CuFeNPs after 16 h dispersion in different solvents at 25°C. (a–f) Fe/Cu ratio at 0, 2, 4, 5, 5.5 and 6, respectively.

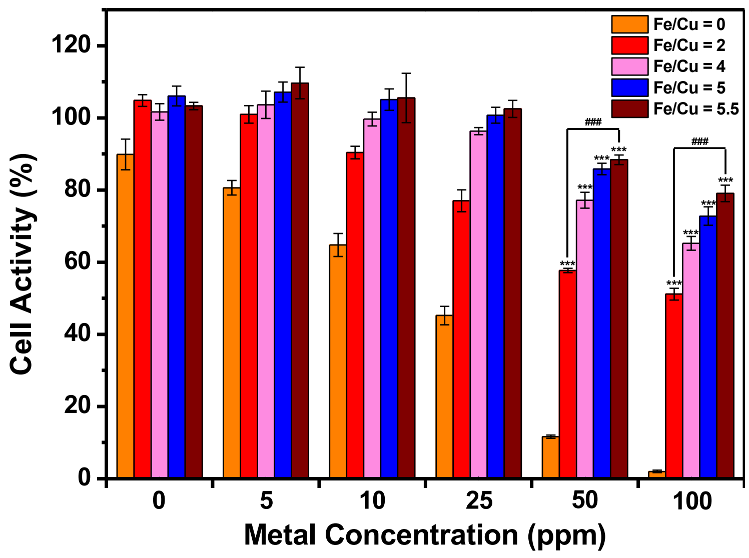

Figure S6. Cell activity of HeLa cells after 24 h co-incubation with CuFeNPs. (n = 4. ***: $p < 0.001$, compared to Fe/Cu = 0. ###: $p < 0.001$, compared between Fe/Cu = 2 and 5.5.)

Table S1. Formula of synthesis of CuFeNPs.

Chemical	Concentration	Fe/Cu ratio of reactant			
		0	0.25	0.5	1
PSMA	240 mg/mL	2.5 mL	2.5 mL	2.5 mL	2.5 mL
DI water	-	7.5 mL	7.375 mL	7.25 mL	7 mL
$CuCl_2$	5 mM	1 mL	1 mL	1 mL	1 mL
$FeCl_2$	10 mM	0 mL	0.125 mL	0.25 mL	0.5 mL
HCl	2 M	18 μL	18 μL	18 μL	18 μL
N_2H_4	64%	100 μL	100 μL	100 μL	100 μL

Table S1. Formula of synthesis of CuFeNPs. (cont'd)

Chemical	Concentration	Fe/Cu ratio of reactant				
		2	4	5	5.5	6
PSMA	240 mg/mL	2.5 mL	2.5 mL	2.5 mL	2.5 mL	2.5 mL
DI water	-	6.5 mL	5.5 mL	5 mL	4.75 mL	4.5 mL
$CuCl_2$	5 mM	1 mL	1 mL	1 mL	1 mL	1 mL
$FeCl_2$	10 mM	1 mL	2 mL	2.5 mL	2.75 mL	3 mL
HCl	2 M	18 μL	18 μL	18 μL	18 μL	18 μL
N_2H_4	64%	100 μL	100 μL	100 μL	100 μL	100 μL

Table S2. Hydrodynamic diameter and zeta potential of CuFeNPs. (n=3)

Fe/Cu ratio	Average hydrodynamic diameter (nm)	PdI	Average zeta potential (mV)
0	102.17 ± 0.66	0.052	-30.27 ± 0.37
2	174.37 ± 1.03	0.037	-33.13 ± 0.31
4	148.97 ± 1.56	0.056	-33.87 ± 0.97
5	162.00 ± 1.64	0.054	-31.70 ± 0.57
5.5	143.70 ± 1.07	0.074	-29.30 ± 0.28
